# Shelf-Life Extension of Refrigerated Turbot (*Scophthalmus maximus*) by Using Weakly Acidic Electrolyzed Water and Active Coatings Containing Daphnetin Emulsions

**DOI:** 10.3389/fnut.2021.696212

**Published:** 2021-07-15

**Authors:** Wenru Liu, Qi Wang, Jun Mei, Jing Xie

**Affiliations:** ^1^College of Food Science and Technology, Shanghai Ocean University, Shanghai, China; ^2^Center for Food Science and Engineering, National Experimental Teaching Demonstration, Shanghai Ocean University, Shanghai, China; ^3^Center of Aquatic Product Processing and Preservation, Shanghai Engineering Research, Shanghai Ocean University, Shanghai, China; ^4^Shanghai Professional Technology Service Platform on Cold Chain Equipment Performance and Energy Saving Evaluation, Shanghai Ocean University, Shanghai, China

**Keywords:** active coating, daphnetin, turbot, quality, shelf-life

## Abstract

This research was to investigate the effect of weakly acidic electrolytic water (WAEW) treatments combining with the locust bean gum (LBG) and sodium alginate (SA) active coatings, containing daphnetin emulsions on microbiological, physicochemical, and sensory changes of turbot (*Scophthalmus maximus*) during refrigerated storage at 4°C for 24 days. Results showed that WAEW, together with LBG-SA coatings containing daphnetin emulsions treatments, could significantly lower the total viable count (TVC), H_2_S-producing bacteria, pseudomonas spp., and psychrotrophic bacteria counts, and inhibit the productions of off-flavor compounds, including the total volatile basic nitrogen (TVB-N), inosine (HxR), and hypoxanthine (Hx). Furthermore, the treatments also prevented textural deterioration, delayed water migration, and had higher organoleptic evaluation results. Therefore, WAEW, together with LBG-SA coatings, containing daphnetin emulsions treatments, had the potential to improve the quality of turbot during refrigerated storage.

## Introduction

Turbot (*Scophthalmusmaximus*) has high economic value and nutritional value and is widely cultivated in China (1). The flavor and high glial protein contents of turbot make it an economically important fish in high demand (2). However, fresh turbot is perishable due to chemical and biological changes, and its organoleptic properties have easily deteriorated during refrigerated storage (3).

The quality deterioration of fish after death results from the microbiological spoilage and biochemical reactions. The specifics spoilage organisms (SSOs) are considered to play a key role in the fish spoilage process (4). Weak acid electrolytic water (WAEW) is produced by the electrolysis of dilute sodium chloride or hydrochloric acid solution and exhibits strong antibacterial activities against SSOs, which has been considered as a new sanitizer (5). WAEW kills the SSOs physically, and it does not generate resistance (6). The antimicrobial mechanism of WAEW may be related to the damage of the microbial cell protective barrier, the changes of the cell membrane permeability, the leakage of inclusions, and the inactivation of some key enzymes (7). WAEW has been tested against the main foodborne pathogens, including *Escherichia coli, Listeria monocytogenes, salmonella*, and *staphylococcus aureus* (8). Palotás et al. (9) reported that a common carp (*Cyprinus carpio*) treated with WAEW (100-mg/kg chloride ion concentration for 5 min) had additional bactericidal efficacy on the surface of the carp fillets and increased the shelf life of the samples, causing 2.4-lg CFU/g decrease, compared with the control by the end of the 7-day storage at 2°C. Khazandi et al. (10) stated that WAEW (either 45 or 150 mg/kg of free chlorine) significantly reduced the total bacterial load and SSOs on King George whiting and Tasmanian Atlantic salmon fillets (about 1–2 lg CFU/g) during storage at 4°C and significantly extended the shelf life of the fillets by 2 and 4 days, respectively.

Active coatings could retard the chemical and microbiological deteriorations of foods by serving as both gas barriers and carriers of food antioxidant or antimicrobial agents (11). The sources of proteins, lipids, or polysaccharides have been researched to meet the demand for novel and environmentally sustainable biomaterials for active coatings (12). Plant-based gums are promising materials for fish preservation because they can keep good quality and prolong the shelf life of foods by increasing a water barrier, reducing microbial contamination, maintaining the flavor, retarding reducing the degree of shrinkage distortion, and preventing fat oxidation (13). However, the active coatings have limitations in antimicrobial and antioxidative properties. The combination of antimicrobial or antioxidative agents with active coatings for fish preservation is of great interest (11). Among natural preservatives, plant polyphenols possess multiple biological functions in inhibiting lipid oxidation, enzymatic reactions, and food spoilage by microorganisms (14). Bazargani-Gilani and Pajohi-Alamoti (15) reported that sodium alginate (SA) active coating containing resveratrol inhibited the increase of pH, peroxide, and K values of rainbow trout fillets and extended the shelf life during refrigerated storage. Cao et al. (16) showed chitosan coating containing chlorogenic acid could inhibit lipid and protein oxidation in snakehead fish fillets stored at 2°C for 5 months. Nie et al. (17) reported the pectin coating infused with gallic acid had lower levels of total volatile basic nitrogen (TVB-N), lipid oxidation, and total sulfhydryls of Japanese sea bass fillets stored at 4°C. Besides, active coatings have been successfully combined, within a hurdle strategy, with other technologies as, for example, WAEW. Luan et al. (18) reported that the chitosan active coating combined with WAEW treatments could retard hairtail spoilage and slow down the protein deterioration of a hairtail (*Trichiutus haumela*) fillet during cold storage at −3°C, which extended the shelf life of hairtail to 6–7 days. Feng et al. (19) stated that active coatings containing epsilon-polylysine hydrochloride and rosemary extract combined with WAEW could effectively inhibit microbial growth and delay the increase in TVB-N, thiobarbituric acid and metmyoglobin value in puffer fish (*Takifugu obscurus*) during refrigerated storage at 4°C, which extended the shelf life to 14 days.

Daphnetin (7, 8-dihydroxycoumarin) is a dihydroxylated derivative of coumarin derived from plants and has been reported to possess antimicrobial, antioxidant, antimalarial, anticoagulation, and immunomodulating activities (20). Despite the fact that there were some studies on daphnetin extending the shelf life of fish during refrigerated storage (21), little is known about the effect of WAEW combining with active coatings containing daphnetin emulsions on fish preservation. Therefore, the objective of the present study was to evaluate the preservative effects of WAEW combining with locust bean gum (LBG) and SA-based active coatings containing different concentrations of daphnetin emulsions (0.16, 0.32, and 0.64 g/L, respectively) on turbot during refrigerated storage at 4°C in the aspects of microbiological analysis, TVB-N,K-values, free amino acids (FAAs), water distribution and migration, texture profile analysis (TPA), and organoleptic evaluation.

## Materials and Methods

### Preparation of Turbot Samples

WAEW was generated by electrolyzing hydrochloric acid solution (3%) in a chamber, using a continuous electrolysis generator (FX-SWS100, Fangxin Water Treatment Equipment Co. Ltd., Yantai, China). The parameters of the WAEW were as follows: the concentration of hypochlorous acid was 30 mg/kg; the oxidation-reduction potential was 1,100 mV, and the pH value was 6.5. The concentrations of daphnetin emulsions used in the present study were 0.16, 0.32, and 0.64 g/L, respectively, and prepared according to our previous research (21). The final active coating solutions were marked as LBG-SA-0.16D, LBG-SA-0.32D, and LBG-SA-0.64D, respectively.

Fresh turbot weighing 500 ± 50 g was purchased from a local market in Luchao Port and randomly divided into five batches: (i) washed with deionized water (CK); (ii) immersed in WAEW for 10 min, followed by a coating with an LBG-SA-active coating (0D); (iii) immersed in WAEW for 10 min, followed by a coating with an LBG-SA-0.16D-active coating (0.16D); (iv) immersed in WAEW for 10 min, followed by a coating with an LBG-SA-0.32D-active coating (0.32D); (vi) and immersed in WAEW for 10 min, followed by a coating with an LBG-SA-0.64D-active coating (0.64D). Then the treated turbot samples were individually packed in a sterile polyethylene bag and stored at 4°C. Turbot samples were randomly sampled for quality analysis on 0, 4, 8, 12, 16, 20, and 24th days, respectively.

### Microbiological Analysis

About 5-g turbot samples and 45-ml normal saline were fully homogenized and subjected to serial dilutions. The microbiological analyses were carried out (22): (i) determination of total viable counts (TVC) on a plate count agar medium was cultivated at 30°C for 48 h; (ii) determination of H_2_S-producing bacteria on an iron agar medium was cultivated at 30°C for 48 h; (iii) determination of *Pseudomonas* spp. on a *Pseudomonas* CFC selective agar medium was cultivated at 30°C for 48 h; (iv) determination of psychrophilic bacteria on a plate count agar medium was cultivated at 4°C for 7 days. The final calculation result was the logarithm of the mean of colony forming units (CFU) on the culture medium with 30–300 colonies.

### Determination of TVB-N

TVB-N determination was performed with the method of Zhuang et al. (23). In brief, 5.0-g minced turbot flesh was homogenized with 45-ml deionized water. The homogenate was stirred at 25°C for 30 min, and then centrifuged at 3,040 × g for 5 min. Subsequently, 5.0 ml of the supernatant was taken to determine the TVB-N content, using the Kjeldahl nitrogen-determination instrument (Kjeltec 8,400, Foss, Denmark). TVB-N content was expressed as a mg N/100-g turbot sample.

### Determination of K Value

The ATP-related compounds were measured by HPLC (Waters 2,695, Milford, CT, USA) according to Cao et al. (24). The K value was determined according to the following concentration ratio:

$$\begin{array}{l} K\text{value}\%=\frac{\text{HxR}+\text{Hx}}{\text{ATP}+\text{ADP}+\text{AMP}+\text{IMP}+\text{HxR}+\text{Hx}}\;\times \text{100} \end{array}$$

### Determination of FAAs

FAAs were performed as described by Yu et al. (25) using, an amino acid analyzer (Hitachi L-8800, Tokyo, Japan). The FAAs identification and quantification were completed by the retention time and peak area with reference to FAAs standards (Sigma Chemical Co. St Louis, MO).

### Low-Field Nuclear Magnetic Resonance Analysis

The distribution and migration of water in turbot samples were evaluated through proton relaxation experiments, using an LF-NMR analyzer (NiumagMesoMR23-60H.I, Suzhou, China), with a proton resonance frequency of 21 MHz (corresponding to the pulse sequence of Carr–Purcell–Meiboom–Gill) (26). Thedorsal muscle of turbot samples were cut into 3 × 2 × 1.5 cm (about 5 g) and wrapped with polyethylene film. For each measurement, 16 scans were performed with 3,000 echoes.

### Magnetic Resonance Imaging Analysis

The proton density-weighted images were obtained by MRI experiments on all turbot samples, also using the above-mentioned LF-NMR analyzer. The slice width was 1.4 mm, time of repetition was 500 ms, and time of echo was 20 ms.

### Determination of TPA

TPA was performed, using a texture analyzer (TA.XT Plus; Stable Micro Systems, Ltd., Godalming, Surrey, UK), equipped with a cylindrical probe (P/5). The 3 × 2 × 1.5 cm (about 5 g) dorsal muscle was tested with a constant test speed of 1 m/s and sample deformation of 50% to obtain the parameters of hardness, springiness, chewiness, and cohesiveness. Each experiment was repeated six times.

### Organoleptic Evaluation

For organoleptic evaluation, the quality index method (QIM) developed by Meral et al. (27) was mentioned. Ten trained professional panelists participated in the organoleptic evaluation. The odor, color, mucus, elasticity, and muscle tissue of the turbot samples were scored at each sampling time. Number 10 indicates the best quality, while a lower score indicates poor quality. The participants were asked to state whether the turbot sample was acceptable or not to determine the shelf life.

### Statistical Analysis

The one-way ANOVA-Duncan test program in SPSS 22.0 software was used for multiple comparisons, and the results were expressed as means ± SD.

## Results and Discussions

### Microbiological Results

**Figure 1:**
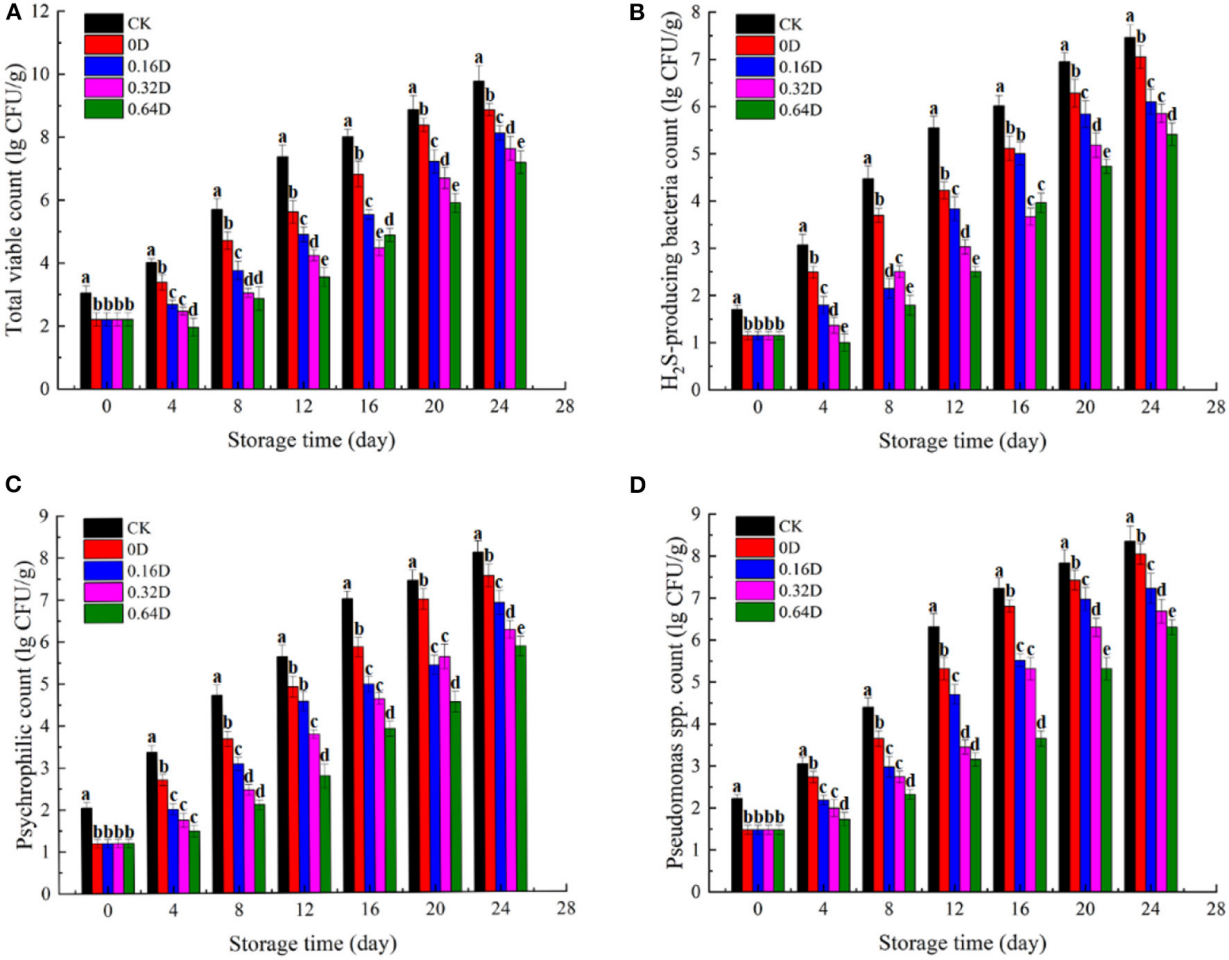
Changes in **(A)** total viable counts, **(B)** H_2_S-producing bacteria counts, **(C)** psychrophilic counts, and **(D)** *Pseudomonas* spp. counts of turbot samples during refrigerated storage [CK, washed with deionized water; 0D, immersed in weakly acidic electrolytic water (WAEW) for 10 min, followed by a coating with a locust bean gum–sodium alginate (LBG-SA)-active coating; 0.16D, immersed in WAEW for 10 min, followed by a coating with an LBG-SA coating with 0.16 g/L daphnetin; 0.32D, immersed in WAEW for 10 min, followed by a coating with an LBG-SA coating with 0.32 g/L daphnetin; 0.64D: immersed in WAEW for 10 min, followed by a coating with an LBG-SA coating with 0.64 g/L daphnetin].

The changes in microbial communities (lg CFU/g) of all turbot samples during refrigerated storage at 4°C were shown in Figure 1. The TVC count of CK sample on 0 day was 3.1 lg CFU/g, and WAEW treatment reduced the TVC count to 2.2 lg CFU/g. The TVC counts of all turbot samples increased during refrigerated storage, and the WAEW and daphnetin-treated samples had lower TVC counts in each sampling time, compared with the CK sample. The TVC count of CK sample on 12th day was 7.4 lg CFU/g, which exceeded the “shelf-life” limit of 7.0 lg CFU/g for marine fish (28). H_2_S-producing bacteria (mainly *Shewanella putrefaciens*) and *Pseudomonas* spp. are the common SSOs in some marine fish and fish products during refrigerated storage (29, 30). The population of the two microbial communities increased with storage time in all samples (Figures 1B,C), which showed similar trends as that of TVC. At the beginning, the counts of H_2_S-producing bacteria of the CK and WAEW-treated samples were 2.7 and 1.1 lg CFU/g, respectively, and the counts increased in all the samples during refrigerated storage. The H_2_S-producing bacteria counts were reached to 7.5, 7.1, 6.1, 5.9, and 5.4 lg CFU/g for CK, 0, 0.16, 0.32, and 0.64D, respectively, at the end of the storage. The initial counts of *Pseudomonas* spp. treated with/without WAEW were 2 and 1.1 lg CFU/g and increased to over 7 lg CFU/g on 16 and 20^th^ days for both CK and 0D samples, respectively. Other samples were still under 7 lg CFU/g at the end of the storage. Psychrotrophic bacteria could cause deterioration in odor, texture and flavor through the production of metabolic compounds, such as aldehydes, ketones, biogenic amines, and volatile sulfides (31). In the current study, the counts of psychrotrophic bacteria treated with/without WAEW were 2.1 and 1.2 lg CFU/g and increased in all the turbot samples during refrigerated storage. The CK and 0D samples exceeded the upper acceptable limit of 6 lg CFU/g on the 16th day; however, the daphnetin-treated turbot samples were still below the upper limit at the end of the storage.

### TVB-N Results

**Figure 2:**
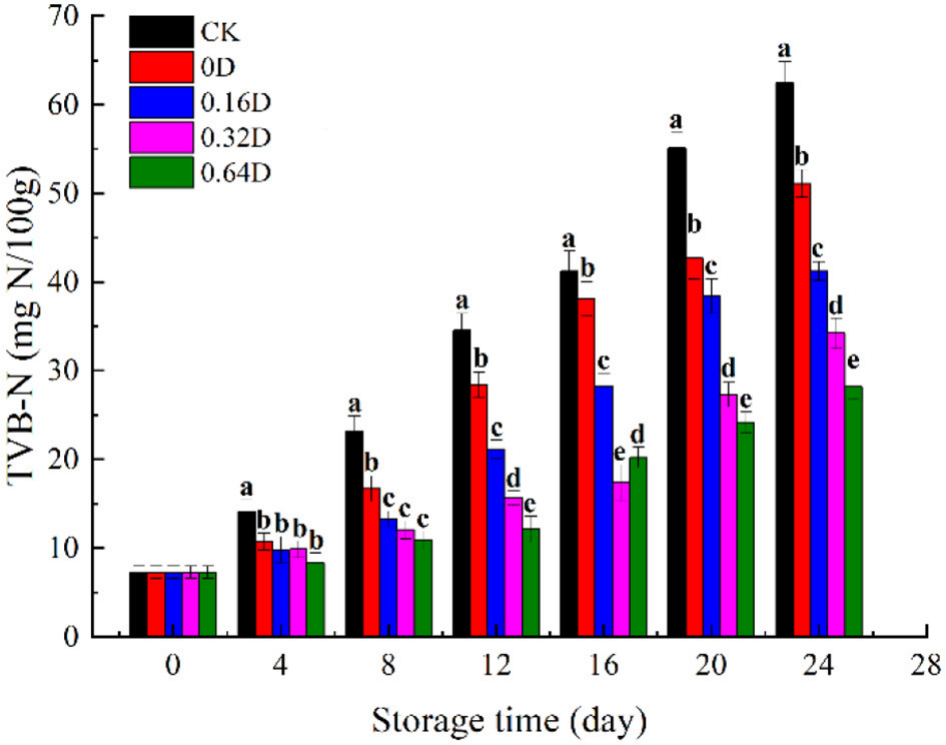
Changes in total volatile basic nitrogen values (TVB-N) of turbot samples during refrigerated storage [CK, washed with deionized water; 0D, immersed in weakly acidic electrolytic water (WAEW) for 10 min, followed by a coating with a locust bean gum–sodium alginate (LBG-SA)-active coating; 0.16D, immersed in WAEW for 10 min, followed by a coating with an LBG-SA coating with 0.16 g/L daphnetin; 0.32D, immersed in WAEW for 10 min, followed by a coating with an LBG-SA coating with 0.32 g/L daphnetin; 0.64D, immersed in WAEW for 10 min, followed by a coating with an LBG-SA coating with 0.64 g/L daphnetin].

The TVB-N quantifies the presence of nitrogenous compounds (ammonia, dimethyl amine, and trimethyl amine) in marine fish, revealing the degree of freshness (32). Its increase during storage is related to the activity of spoilage bacteria and endogenous enzymes (33). The TVB-N values of turbot samples during refrigerated storage are presented in Figure 2. The initial TVB-N value was 7.26 mg N/100 g, indicating good quality and increased with storage time for all the samples; furthermore, the increase was faster after the middle of refrigerated storage because of the increased bacterial activity, endogenous enzymes, storage conditions, and hygienic practices (34). The CK sample had higher TVB-N value throughout the refrigerated storage, and its fits with that TVC had a sharp increase. The CK and 0D samples exhibited a higher increase rate, reaching to 34.58 and 38.61 mg N/100g on 12 and 16^th^ days, exceeded the maximum allowable value (30–35 mg N/100 g) of TVB-N in marine fish (35). The usage of WAEW and daphnetin treatments showed a significant inhibiting effect on the TVB-N increase of turbot samples during the refrigerated storage (*p* < 0.05), compared with the CK sample. The 0.64D sample was still acceptable at the end of the storage, which indicated that WAEW and daphnetin treatments could effectively inhibit the growth of microorganisms and slow down the production of nitrogen and amine substances to delay the deterioration of turbot. Some studies showed that the microbial changes of fish were correlated with TVB-N values (36, 37). Similarly, the TVB-N value of turbot increased with the microbial growth during refrigerated storage, indicating that volatile alkaline compounds were mainly produced by the metabolic activities of SSOs (38). WAEW and daphnetin treatments inhibited the microbial growth in turbot during refrigerated storage, protecting toward protein degradation and producing less ammonia and amine compounds, thus having lower TVB-N values, compared with the CK sample (39).

### K Value Results

**Figure 3:**
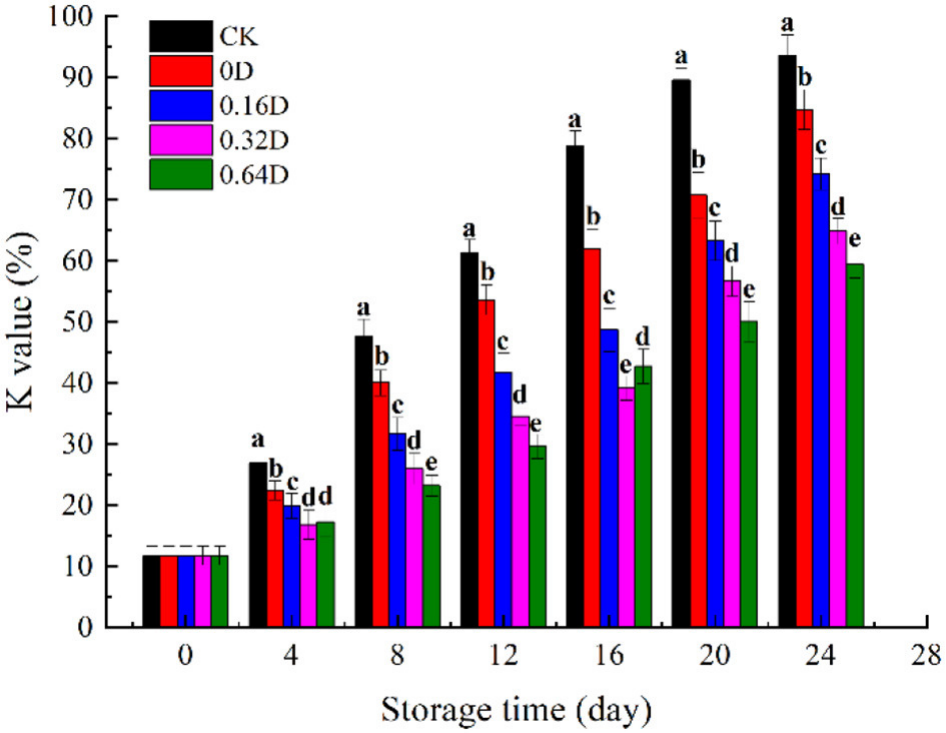
Changes in K value of turbot samples during refrigerated storage [CK, washed with deionized water; 0D, immersed in weakly acidic electrolytic water (WAEW) for 10 min, followed by a coating with a locust bean gum–sodium alginate (LBG-SA)-active coating; 0.16D, immersed in WAEW for 10 min, followed by a coating with an LBG-SA coating with 0.16 g/L daphnetin; 0.32D, immersed in WAEW for 10 min, followed by a coating with an LBG-SA coating with 0.32 g/L daphnetin; 0.64D, immersed in WAEW for 10 min, followed by a coating with an LBG-SA coating with 0.64 g/L daphnetin].

The K value has been useful for determining quality as it negatively correlates with the freshness of fish, and the increase in K-value is related to the degradation of ATP (40). The degradation of ATP is an autolytic change, which is accompanied by muscle softening during the stiffness process and results in a decrease in fish freshness. The sequence of ATP degradation to Hx is as follows: ATP → ADP → AMP → IMP → HxR → Hx (41). The initial K value was found to be 11.72% (Figure 3), and the muscle of turbot is fresh, which was similar to the previously reported value of turbot (42). The K value increased in all samples during refrigerated storage, and the CK and 0D samples increased significantly faster than that of the daphnetin-treated sample (*p* < 0.05). The K value of CK 0, 0.16, 0.32, and 0.64D samples at the end of storage were 93.61%, 84.72%, 74.16%, 64.85%, and 59.36%, respectively, where the K values of the 0.64D-treated samples were significantly lower than the other samples. Only the 0.64D sample was still acceptable (lower than 60%), and other samples were categorized as fish spoilage (>60%) (43). WAEW and daphnetin treatments could effectively reduce the degradation of ATP, which can be attributed to the inhibitory effect of WAEW and daphnetin on nucleotide-degrading enzymes and bacteria responsible for nucleotide degradation (44). As reported by Jung et al. (45), the brown sole (*Pleuronectes herzensteini*) treated with WAEW could delay the degradation of nucleic acids-related substances in the muscle and retain the freshness of the fish. According to Alasalvar et al. (46), the conversion of ATP to IMP was a totally autolytic process within 1–2-day storage; however, the subsequent breakdown of IMP to Hx was caused by both fish and microbial enzymes. In the present research, the decreased degradation of ATP to IMP in WAEW and daphnetin-treated samples might result from inactivation of microorganisms by both WAEW and LBG-SA active coatings, containing daphnetin emulsions.

### FAAs Results

FAAs are the main contributors to the development of taste sensations in fish, including umami, bitterness, and sweetness, as well as being precursors to biogenic amines, such as histamine derived from histidine *via* microbial metabolism (47), which could affect the flavor and taste of aquatic products during processing or storage. Alanine and glycine have a sweet taste (48), while glutamic acid and aspartic acid have the typical “freshness” of aquatic products (3). The concentrations of FAAs in turbot samples on 0, 12, and 24th days are shown in Table 1. In the present research, most of FAAs and total FAAs showed upward trends in all the samples during the refrigerated storage. The main FAAs in the turbot samples were glutamic acid, glycine, alanine, valine, phenylalanine, and lysine, accounting for 49.23–55.49% of the total FAAs contents. The glycine content in the CK samples increased from 5.12 mg/100 g on 0 day to 14.56 mg/100 g at the end of storage. The changes of glycine content in the WAEW and daphnetin-treated samples were similar to that of the CK samples; however, their final contents were significantly (*p* < 0.05) higher. The glutamic acid and alanine contents of WAEW and daphnetin-treated turbot samples also increased significantly during the refrigerated storage due to the short peptides were broken down by proteolytic enzymes, resulting in an increase in the FAAs contents. Histidine, as an off-flavor amino acid, accounted for 1.22–11.99% of the total FAAs contents. The histidine content in the CK samples increased from 1.57 mg/100 g on 0 day to 32.02 mg/100 g at the end of storage; however, the histidine contents of 0.16, 0.32, and 0.64D samples were 16.46, 10.48, and 14.50 mg/100 g at that time, respectively. The lower histidine concentration resulted in a reduction of bitterness in the turbot samples during the refrigerated storage (47). The flavor deterioration is associated with the reduction of some umami and sweet flavor enhancing amino acids and the accumulation of off-flavor amino acids. WAEW and LBG-SA active coatings, containing daphnetin emulsions treatments, were effective in delaying the flavor deterioration process and maintaining the flavor of turbot samples during the refrigerated storage.

**Table 1:** Changes in the free amino acid content of turbot during the refrigerated storage at 4°C.

**Storage Time**	**Samples**	**FAAs**								
		**Asp**	**Thr**	**Ser**	**Glu**	**Gly**	**Ala**	**Cys**	**Val**	**Met**
0d	-	0.46 ± 0.01	1.99 ± 0.05	4.40 ± 0.13	3.70 ± 0.11	5.12 ± 0.18	8.31 ± 0.25	1.46 ± 0.02	5.11 ± 0.15	2.27 ± 0.10
12d	CK	1.87 ± 0.03^b^	3.75 ± 0.02^b^	2.73 ± 0.09^c^	5.38 ± 0.08^d^	6.46 ± 0.08^c^	18.37 ± 0.23^c^	1.70 ± 0.01^a^	7.33 ± 0.12^b^	3.32 ± 0.08^b^
	0D	1.76 ± 0.02^c^	3.77 ± 0.01^b^	5.79 ± 0.16^b^	7.89 ± 0.14^b^	5.96 ± 0.05^d^	19.10 ± 0.05^b^	1.54 ± 0.01^c^	6.62 ± 0.01^c^	2.89 ± 0.12^c^
	0.16D	1.28 ± 0.02^d^	3.62 ± 0.05^bc^	5.85 ± 0.15^b^	6.66 ± 0.16^c^	7.44 ± 0.18^a^	13.89 ± 0.27^e^	1.57 ± 0.01^bc^	6.31 ± 0.14^cd^	2.38 ± 0.05^d^
	0.32D	0.98 ± 0.01^e^	3.38 ± 0.10^c^	5.59 ± 0.03^b^	7.94 ± 0.07^b^	5.31 ± 0.03^e^	16.26 ± 0.06^d^	1.60 ± 0.01^b^	6.06 ± 0.04^d^	3.34 ± 0.01^b^
	0.64D	2.66 ± 0.02^a^	5.57 ± 0.06^a^	6.47 ± 0.06^a^	10.42 ± 0.19^a^	7.00 ± 0.27^b^	20.48 ± 0.10^a^	-	8.84 ± 0.03^a^	5.29 ± 0.02^a^
24d	CK	1.99 ± 0.01^e^	2.62 ± 0.01^e^	-	9.75 ± 0.10^e^	14.56 ± 0.03^e^	32.48 ± 0.28^d^	1.34 ± 0.01^e^	25.33 ± 0.19^c^	6.45 ± 0.23^e^
	0D	2.75 ± 0.01^c^	11.04 ± 0.02^c^	-	17.56 ± 0.01^b^	21.86 ± 0.02^b^	46.30 ± 0.06^a^	3.76 ± 0.01^b^	35.32 ± 0.04^a^	26.45 ± 0.05^c^
	0.16D	2.24 ± 0.02^d^	22.69 ± 0.06^a^	-	15.44 ± 0.03^d^	17.84 ± 0.04^d^	43.34 ± 0.09^b^	3.02 ± 0.01^c^	33.81 ± 0.07^b^	28.30 ± 0.06^b^
	0.32D	2.84 ± 0.06^b^	3.06 ± 0.02^d^	1.21 ± 0.03	22.22 ± 0.03^a^	24.31 ± 0.05^a^	38.61 ± 0.12^c^	9.92 ± 0.06^a^	34.93 ± 0.18^a^	31.88 ± 0.17^a^
	0.64D	4.73 ± 0.04^a^	19.95 ± 0.28^b^	-	15.88 ± 0.18^c^	20.99 ± 0.23^c^	43.97 ± 0.48^b^	2.32 ± 0.03^d^	33.65 ± 0.31^b^	23.27 ± 0.14^d^
		Ile	Leu	Tyr	Phe	Lys	His	Arg	Pro	Total
0d	-	2.47 ± 0.07	3.74 ± 0.10	1.82 ± 0.02	2.33 ± 0.03	0.78 ± 0.04	1.57 ± 0.07	2.15 ± 0.08	3.06 ± 0.14	50.73 ± 1.50
12d	CK	3.04 ± 0.04^b^	4.55 ± 0.06^bc^	3.16 ± 0.06^b^	3.11 ± 0.03^b^	0.78 ± 0.01^a^	2.32 ± 0.04^a^	2.85 ± 0.05^d^	4.54 ± 0.10^b^	75.26 ± 1.09^b^
	0D	3.13 ± 0.12^b^	4.32 ± 0.08^c^	3.18 ± 0.07^b^	2.25 ± 0.07^b^	0.27 ± 0.03^b^	2.39 ± 0.06^a^	3.03 ± 0.06^c^	4.92 ± 0.09^b^	78.84 ± 1.30^b^
	0.16D	3.05 ± 0.06^b^	4.50 ± 0.08^bc^	2.69 ± 0.03^bc^	3.10 ± 0.04^b^	0.29 ± 0.01^b^	2.19 ± 0.05^a^	3.23 ± 0.10^b^	8.50 ± 3.31^a^	76.56 ± 2.94^b^
	0.32D	3.31 ± 0.03^b^	4.96 ± 0.05^b^	2.69 ± 0.03^bc^	3.24 ± 0.03^b^	1.00 ± 0.15^a^	2.11 ± 0.08^a^	3.60 ± 0.04^a^	8.02 ± 3.47^a^	79.38 ± 3.90^b^
	0.64D	5.34 ± 0.01^a^	8.46 ± 0.03^a^	7.09 ± 0.13^a^	7.65 ± 0.12^a^	0.85 ± 0.01^a^	1.25 ± 0.04^b^	1.44 ± 0.03^e^	3.66 ± 0.05^c^	102.46 ± 0.50^a^
24d	CK	17.06 ± 0.18^d^	31.31 ± 0.20^e^	26.09 ± 0.21^e^	40.41 ± 0.38^d^	21.63 ± 0.23^e^	32.02 ± 0.48^a^	1.16 ± 0.01^e^	2.93 ± 0.01^c^	267.13 ± 2.24^e^
	0D	22.02 ± 0.01^b^	40.77 ± 0.05^c^	29.12 ± 0.80^d^	41.83 ± 0.62^c^	43.45 ± 0.09^b^	22.48 ± 0.02^b^	1.82 ± 0.07^c^	5.33 ± 0.17^b^	371.83 ± 1.52^c^
	0.16D	21.68 ± 0.07^c^	42.35 ± 0.16^b^	48.16 ± 0.28^b^	60.76 ± 0.33^b^	44.13 ± 0.24^a^	16.46 ± 0.65^c^	2.05 ± 0.01^b^	6.76 ± 3.38^a^	409.03 ± 2.26^a^
	0.32D	22.54 ± 0.11^a^	43.57 ± 0.22^a^	60.74 ± 0.70^a^	62.58 ± 0.57^a^	23.86 ± 0.20^c^	10.48 ± 0.01^e^	1.34 ± 0.01^d^	2.63 ± 0.10^c^	396.72 ± 2.34^b^
	0.64D	22.01 ± 0.25^b^	39.01 ± 0.42^d^	34.37 ± 0.51^c^	38.83 ± 0.45^e^	22.91 ± 0.23^d^	14.50 ± 0.17^d^	2.57 ± 0.03^a^	5.24 ± 0.13^b^	344.20 ± 3.45^d^

“*-” is not detected. Different lowercase letters in different groups from the same day indicate significant differences (p < 0.05)*.

### Water Distribution Results

**Figure 4:**
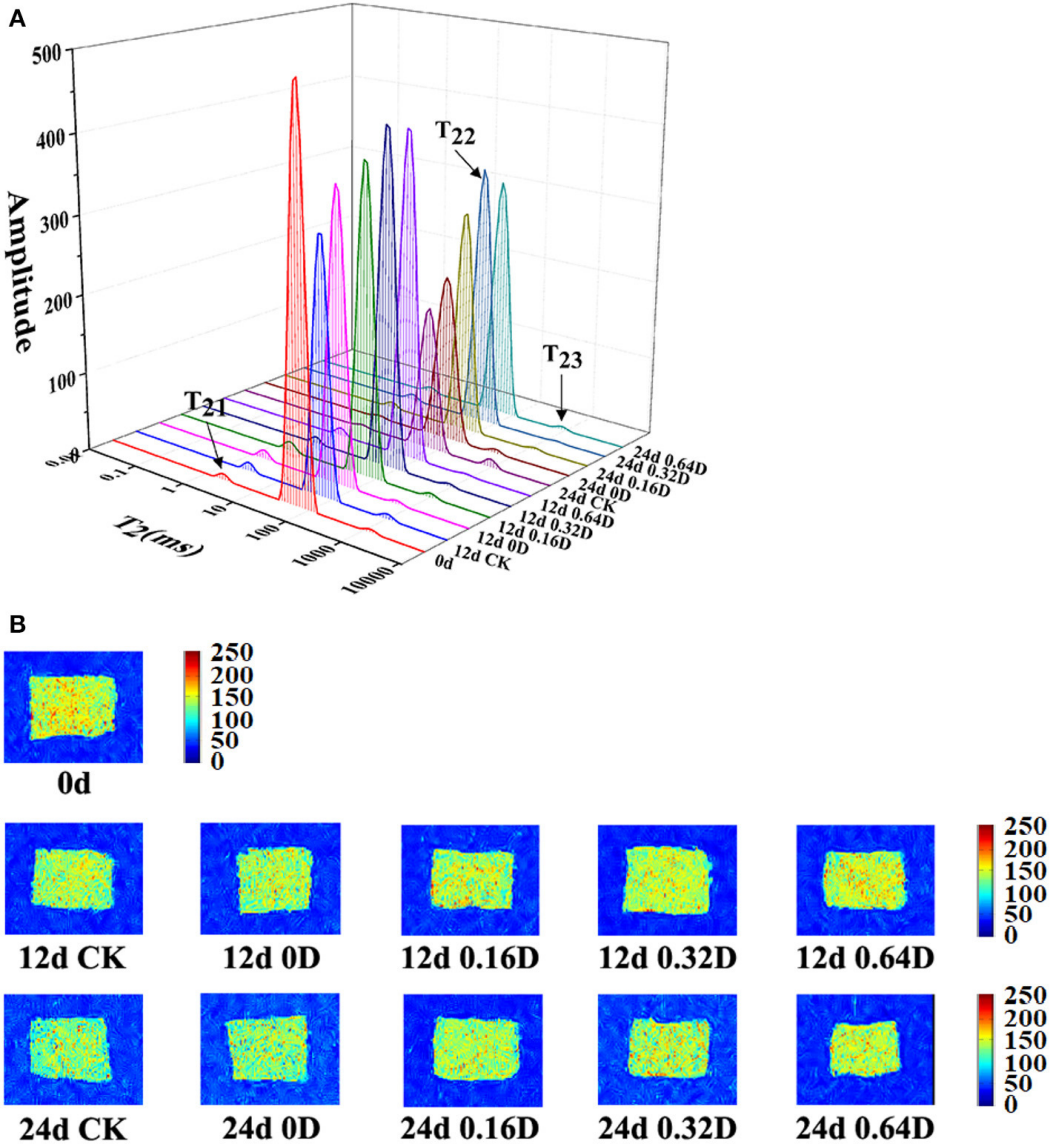
Changes in **(A)** water distribution and **(B)** magnetic resonance imaging of turbot samples during the refrigerated storage [CK, washed with deionized water; 0D, immersed in weakly acidic electrolytic water (WAEW) for 10 min, followed by a coating with a locust bean gum–sodium alginate (LBG-SA)-active coating; 0.16D, immersed in WAEW for 10 min, followed by coating with LBG-SA coating with 0.16 g/L daphnetin; 0.32D, immersed in WAEW for 10 min, followed by coating with LBG-SA coating with 0.32 g/L daphnetin; 0.64D, immersed in WAEW for 10 min, followed by coating with LBG-SA coating with 0.64 g/L daphnetin].

LF-NMR can be used to describe the changes of water distribution and transfer in fish and fish products by measuring the proton relaxation (49). Figure 4A shows the distribution of T_2_ transverse relaxation times of turbot samples on 0, 12, and 24th days during the refrigerated storage. There are three peaks corresponding to the three relaxation components, known as T_21_ (<10 ms), T_22_ (20–400 ms) and T_23_ (>1,000 ms) and stand for bound water, immobilized water, and free water, respectively. The pT_21_, pT_22_, and pT_23_ corresponded to the areas of T_21_, T_22_, and T_23_ (50). pT21 varied, ranging from.83 to 1.12% during the refrigerated storage. There was no significant difference among WAEW and daphnetin-treated samples (*p* > 0.05) in the pT21 during storage, indicating T_21_ could not be affected by WAEW and daphnetin treatments as well as storage time, which was due to the water entrapped within highly organized myofibril structures (51). pT_22_ diminished progressively, and pT_23_ increased constantly during the storage (*p* < 0.05). In the present study, The CK samples had significantly lower immobilized water contents than that of WAEW and daphnetin-treated samples during the refrigerated storage. About 0.32 and 0.64D samples had higher contents of immobilized water on 12 and 24th days, probably owing to the WAEW, and LBG-SA active coatings, containing 0.32 or 0.64 g/L daphnetin emulsions treatments, could effectively suppress the changes of the immobilized water into free water. Some research reported that water located within myofibrillar macromolecules released or translated to free water due to the destruction of myofibril structures (38, 52). Besides, this process of water migration was also well reflected in a followed phenomenon that WAEW and daphnetin treatments retarded the change rates of T_22_ and T_23_. WAEW and daphnetin treatments could effectively delay the water located within myofibrillar macromolecules to release or translate to free water based on the destruction of myofibril structures.

MRI can highlight the signal of different phases of water in the organization. The proton density map is much brighter, and the pseudo-color image is redder (53). The sample was brighter and redder in images on 0 day (Figure 4), and the brightness was darker and bluer with the refrigerated storage time increasing. The color of CK samples on 12 and 24th days was bluer and darker than that of WAEW and daphnetin-treated samples. The results demonstrated that the density of water protons in CK was the lowest compared with the other samples, and the tissue structure was seriously damaged, resulting in water loss. However, there was less structural disruption of myofibrils in WAEW and daphnetin-treated samples during the refrigerated storage (54). The brightness of 0.32D and 0.64D samples was lighter compared with the other samples, which indicated that the WAEW and LBG-SA active coatings, containing 0.32 or 0.64-g/L daphnetin emulsions treatments could be more suitable for quality maintenance of turbot samples during refrigerated storage, and the result was consistent with the variation of LF-NMR transverse relaxation.

### TPA Results

**Figure 5:**
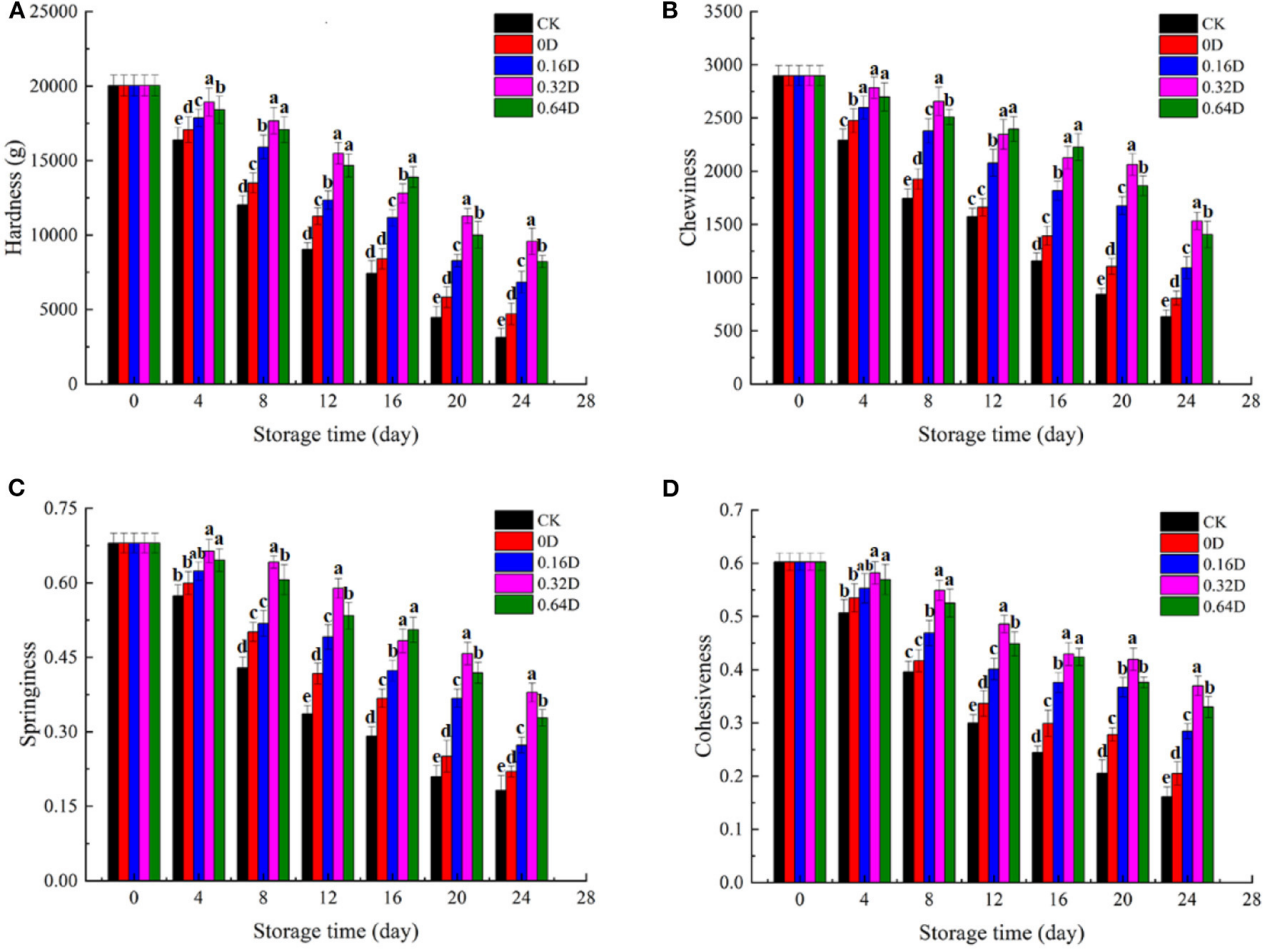
Changes in **(A)** hardness, **(B)** chewiness, **(C)** springiness, and **(D)** cohesiveness of turbot samples during the refrigerated storage [CK, washed with deionized water; 0D, immersed in weakly acidic electrolytic water (WAEW) for 10 min, followed by coating with locust bean gum–sodium alginate (LBG-SA)-active coating; 0.16D, immersed in WAEW for 10 min, followed by coating with LBG-SA coating with 0.16-g/L daphnetin; 0.32D, immersed in WAEW for 10 min, followed by coating with LBG-SA coating with 0.32 g/L daphnetin; 0.64D, immersed in WAEW for 10 min, followed by coating with LBG-SA coating with 0.64-g/L daphnetin].

Texture is considered as a valuable quality attribute to evaluate the influence of preservation (55). The physical properties (hardness, chewiness, springiness, and cohesiveness) for textural evaluation of turbot samples during the refrigerated storage are shown in Figure 5. The texture properties of all the turbot samples were decreased during the refrigerated storage, indicating the Argo turbot samples lost good quality and freshness (5). The hardness of the fresh sample was 2.0 × 10^4^ g and decreased significantly (*p* < 0.05), with increasing storage time, regardless of the storage conditions. The muscles became softer and less elastic with increasing storage time, resulting from the activity of the autolytic enzyme, the degradation of myofibrils, and the destruction of the connective tissue (56). The chewiness showed similar behaviors with that of hardness as a decrease in these two parameters was observed during the refrigerated storage, which is consistent with the findings of Liu et al. (57). WAEW and daphnetin-treated samples had significant higher chewiness values (*p* < 0.05) compared with the CK samples during the storage. Springiness is used to describe the ability of muscles to return to their original form after the removal of deformation force, as well as their resistance to subsequent deformation (58). The average value of springiness of fresh turbot was about.67 and decreased significantly (*p* < 0.05) by 73.19, 67.60, 59.80, 44.23, and 51.68% for CK, 0, 0.16, 0.32, and 0.64D turbot samples at the end of storage, respectively, indicating the occurrence of deterioration in texture. Cohesiveness reflects the interior adhesive force of samples. The cohesiveness of turbot also showed a decreasing trend during the refrigerated storage. The initial values of cohesiveness were about 0.60 and decreased to 0.16, 0.20, 0.28, 0.37, and 0.33 for CK, 0D, 0.16D, 0.32D, and 0.64D turbot samples at the end of the storage, respectively. Decrease in springiness and cohesiveness may be attributed to the destruction of covalent cross-linking structure between proteins, the decomposition of proteins, and the reduced force between muscle fiber as the microbial growth and enzymatic carried by the fish itself (59). Some studies pointed out that texture deterioration might be caused by the degradation and disintegration of myofibril structure, affecting the acceptance of consumers (60). The combined effect of WAEW and daphnetin could inhibit the growth of microorganisms and delay the degradation of muscle fibers to protect the muscle texture of turbot. Therefore, WAEW and daphnetin treatments were effective in minimizing changes in turbot muscle tissues during the refrigerated storage.

### Organoleptic Evaluation Results

**Figure 6:**
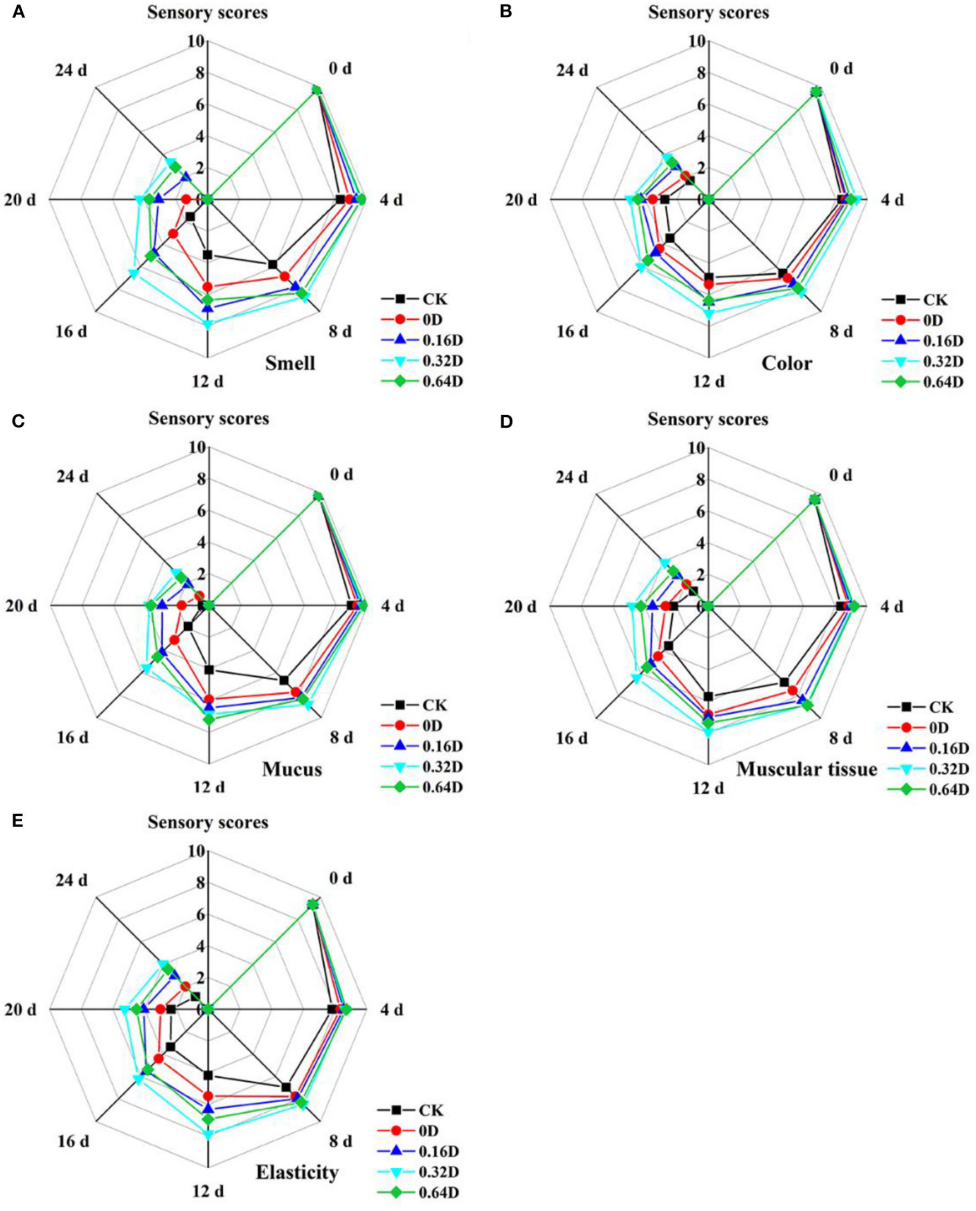
Changes in **(A)** smell, **(B)** color, **(C)** mucus, **(D)** muscular tissue, and **(E)** elasticity of the turbot samples during the refrigerated storage [CK, washed with deionized water; 0D, immersed in weakly acidic electrolytic water (WAEW) for 10 min, followed by coating with locust bean gum–sodium alginate (LBG-SA)-active coating; 0.16D, immersed in WAEW for 10 min, followed by coating with LBG-SA coating with 0.16-g/L daphnetin; 0.32D, immersed in WAEW for 10 min, followed by coating with LBG-SA coating with 0.32 g/L daphnetin; 0.64D, immersed in WAEW for 10 min, followed by coating with LBG-SA coating with 0.64-g/L daphnetin].

The organoleptic evaluation results, including smell, color, mucus, muscular tissue and elasticity of turbot samples during the refrigerated storage, are presented in Figure 6. At the beginning, all the turbot samples had high organoleptic scores, demonstrating excellent quality. The organoleptic scores of all the turbot samples decreased significantly (*p* < 0.05) during the refrigerated storage; however, WAEW and daphnetin-treated samples had significant higher scores (*p* < 0.05) than that of CK samples. On the 12th day, the score of CK was lower than the limit value of 5, which was considered as unacceptable for turbot in the present research. However, 0.16, 0.32, and 0.64D exceeded the limitation on the 16, 20, and 20th days, respectively. WAEW and daphnetin treatments helped the turbot samples maintain a relatively better organoleptic quality during the refrigerated storage; thus, the way of WAEW and daphnetin treatments could be an effective way to delay the quality deterioration and maintain the organoleptic quality of turbot samples. However, the organoleptic evaluation showed some mismatching in the microbiological and chemical results of turbot samples, which may be probably due to the spoilage caused by microbial growth on the surface of the turbot samples. Therefore, it is appropriate to evaluate the storage quality of the turbot samples by the comprehensive analysis of physicochemical, microbiological, and organoleptic evaluation.

## Conclusions

This research investigated the effect of WAEW combined with LBG-SA-active coatings, containing daphnetin emulsions (0.16, 0.32, and 0.64 mg/mL, respectively) treatments on the quality of the turbot samples during the refrigerated storage at 4°C. The microbiological analysis showed that WAEW and daphnetin treatments effectively inhibit the growth of spoilage microorganisms, attributing to the sterilizate activity of WAEW and the antibacterial activity of an active coating, containing daphnetin emulsions. WAEW and daphnetin treatments were highly efficient in maintaining lower TVB-N and K values and higher contents of aromatic amino acids, such as glutamic acid, glycine, and alanine in the refrigerated turbot samples. Moreover, TPA and LN-NMR results also stated that the presence of WAEW combined with daphnetin treatments showed positive effects on retarding the degradation of myofibril structure. WAEW, together with LBG-SA active coatings, containing 0.32 or 0.64-mg/mL daphnetin emulsions treatments had similar effects on quality maintenance of the turbot samples during the refrigerated storage. Therefore, 0.32-mg/mL daphnetin addition was determined to maintain the quality of turbot for economic consideration and the principle of using as few food additives as possible.

## Data Availability Statement

The original contributions presented in the study are included in the article/supplementary material, further inquiries can be directed to the corresponding author/s.

## Author Contributions

WL and JM: conceptualization, investigation, and writing–original draft. WL: data curation, formal analysis, and software. QW: Revising and editing the article. JX: funding acquisition and validation. WL, JM, and JX: methodology. JM and JX: project administration and writing–review & editing. All authors contributed to the article and approved the submitted version.

## Conflict of Interest

The authors declare that the research was conducted in the absence of any commercial or financial relationships that could be construed as a potential conflict of interest.
